# Distinct amyloid and tau PET signatures are associated with diverging clinical and imaging trajectories in patients with amnestic syndrome of the hippocampal type

**DOI:** 10.1038/s41398-021-01628-9

**Published:** 2021-09-29

**Authors:** Julien Lagarde, Pauline Olivieri, Matteo Tonietto, Philippe Gervais, Claude Comtat, Fabien Caillé, Michel Bottlaender, Marie Sarazin

**Affiliations:** 1 Department of Neurology of Memory and Language, GHU Paris Psychiatrie & Neurosciences, Paris, France; 2 Université de Paris, Paris, France; 3 Université Paris-Saclay, BioMaps, Service Hospitalier Frederic Joliot CEA, CNRS, Inserm, Orsay, France; 4 Université Paris-Saclay, UNIACT, Neurospin, Joliot Institute, CEA, Gif sur Yvette, France

**Keywords:** Diagnostic markers, Hippocampus

## Abstract

We aimed to investigate the amyloid and tau PET imaging signatures of patients with amnestic syndrome of the hippocampal type (ASHT) and study their clinical and imaging progression according to their initial PET imaging status. Thirty-six patients with a progressive ASHT and 30 controls underwent a complete neuropsychological assessment, 3 T brain MRI, [^11^C]-PiB and [^18^F]-Flortaucipir PET imaging. Subjects were clinically followed-up annually over 2 years, with a second 3 T MRI (*n* = 27 ASHT patients, *n* = 28 controls) and tau-PET (*n* = 20 ASHT patients) at the last visit. At baseline, in accordance with the recent biological definition of Alzheimer’s disease (AD), the AD PET signature was defined as the combination of (i) positive cortical amyloid load, and (ii) increased tau tracer binding in the entorhinal cortices and at least one of the following regions: amygdala, parahippocampal gyri, fusiform gyri. Patients who did not meet these criteria were considered to have a non-AD pathology (SNAP). Twenty-one patients were classified as AD and 15 as SNAP. We found a circumscribed tau tracer retention in the entorhinal cortices and/or amygdala in 5 amyloid-negative SNAP patients. At baseline, the SNAP patients were older and had lower *ApoE* ε4 allele frequency than the AD patients, but both groups did not differ regarding the neuropsychological testing and medial temporal lobe atrophy. During the 2-year follow-up, the episodic memory and language decline, as well as the temporo-parietal atrophy progression, were more pronounced in the AD sub-group, while the SNAP patients had a more pronounced progression of atrophy in the frontal lobes. Longitudinal tau tracer binding increased in AD patients but remained stable in SNAP patients. At baseline, distinct amyloid and tau PET signatures differentiated early AD and SNAP patients despite identical cognitive profiles characterized by an isolated ASHT and a similar degree of medial temporal atrophy. During the longitudinal follow-up, AD and SNAP patients diverged regarding clinical and imaging progression. Among SNAP patients, tau PET imaging could detect a tauopathy restricted to the medial temporal lobes, which was possibly explained by primary age-related tauopathy.

## Introduction

Progressive amnesia is the core feature of typical Alzheimer’s disease (AD) and can be assessed by different tests of episodic memory. A specific episodic memory disorder has been reported in AD, characterized by a low free recall not normalized with semantic cueing in tests controlling for a successful encoding. In early AD, this amnestic profile is correlated with hippocampal atrophy and gray matter loss in the medial temporal lobe, even at a prodromal stage [1], as well as with AD pathology determined by cerebrospinal fluid (CSF) AD-biomarkers [2]. It is supposed to reflect the hippocampal dysfunction, which underlies the so-called amnestic syndrome of the hippocampal type (ASHT) [3]. The presence of an ASHT was proposed as a core clinical marker of typical AD in the recommendations of the International Working Group (IWG), but it must be supported by AD pathophysiological markers to make a diagnosis, especially at an early stage. Whether the amnestic syndrome of the hippocampal type is a signature of AD pathology has been questioned in a recent neuropathological study, showing that free and cued memory assessment lack accuracy to predict AD pathology [4].

AD is indeed far from being the only cause of progressive amnesia [5, 6], and the biological heterogeneity of this clinical phenotype has been highlighted [7]. Neuropathological studies have also emphasized that different pathological lesions other than AD can lead to episodic memory deficits mimicking AD [4]. The term SNAP, or “suspected non-AD pathophysiology” [8–10] was proposed to identify these individuals, reflecting the notion that pathologies outside of AD (and particularly of amyloid) underlie their neurodegenerative change. The latest version of the National Institute on Aging and the Alzheimer’s Association (NIA-AA) criteria for AD diagnosis defined non-AD pathologic change as the absence of both amyloid and tau lesions [11]. Among non-AD pathologic changes, beyond Lewy body disease, frontotemporal lobar degeneration (FTLD) or vascular lesions, the focus has recently shifted to Hippocampal Sclerosis (HS) in advanced age and limbic-predominant age-related TDP-43 encephalopathy (LATE), associated or not with HS [12, 13]. Tau pathology restricted or predominating in limbic regions has also been described in aged individuals and called primary age-related tauopathy (PART) [14]. Little is known about the neuropsychological and behavioural characteristics of these different entities. In vivo detection of “SNAP” post-mortem diagnoses remains challenging [4] but is crucial because these patients could have different clinical evolutions [15–17] and distinct underlying biological mechanisms, leading to different therapeutic strategies.

Molecular imaging by positron emission tomography (PET) is useful for the in vivo detection of certain proteinopathies. Beyond amyloid PET imaging, in vivo detection of tau pathology is now possible [18]. [^18^F]-Flortaucipir (formerly called AV-1451) binds selectively to tau lesions composed primarily of paired helical filaments, such as intra- and extra-neuronal tangles and dystrophic neuritis [19, 20]. In typical AD, patterns of tracer retention corresponded well with Braak staging of neurofibrillary tau pathology [21] and correlated with the clinical symptoms [22]. The combination of amyloid and tau PET imaging in amnestic patients permits the identification of the pathophysiological process and could limit the risk of misdiagnosis [4, 23, 24].

Very few studies confronted detailed cognitive characterization of patients with progressive amnesia to the in vivo assessment of both amyloid and tau pathologies. To our knowledge, only two studies published by the same team used both amyloid and tau PET imaging in order to identify SNAP [24, 25] in small groups of amnestic patients. None of them provided information about individual tau regional binding within the medial temporal lobes. They did not include longitudinal imaging data, and did not use a strict definition of progressive amnesia as defined above (ASHT).

In the present study, we aimed to determine the amyloid and tau PET signatures in a group of well-characterized patients with ASHT. First, we aimed to (a) differentiate patients with SNAP from patients with early AD according to PET imaging, (b) compare their neuropsychological-behavioural performances and brain atrophy patterns at baseline, (c) analyse individual tau PET regional binding in order to capture possible “PART” within the SNAP group. Second, we aimed at investigating the longitudinal progression of the cognitive deficit, the regional cortical atrophy (including the hippocampus, entorhinal cortex, amygdala and insula) and the tau PET tracer uptake after 2 years of follow-up in SNAP and AD patients. We hypothesized that we could identify a significant proportion of SNAP among these patients with ASHT, and that the distinct molecular signatures (AD vs SNAP) could be associated with diverging clinical and imaging trajectories during longitudinal follow-up, with a more pronounced cognitive decline and progression of temporo-parietal atrophy in AD.

## Materials and methods

### Participants

We prospectively included 66 participants from the Shatau7-Imatau (NCT02576821) and Imabio3 (NCT01775696) studies. The Ethics Committee (Comité de Protection des Personnes Ile-de-France VI) approved the studies. All subjects provided written informed consent.

Thirty-six patients with ASHT (age = 72.9 ± 7.8 years, MMSE = 24.5 ± 2.8) were enrolled according to the following criteria: (i) progressive episodic memory impairment characterized by a low free recall not normalized with semantic cueing (free recall score ≤ 17/48 and/or total recall score ≤ 42/48 at the Free and Cued Selective Reminding Test (FCSRT)) [26]; (ii) no other significant cognitive deficit than executive and social cognition impairments (preservation of language, praxis or visuo-spatial abilities); (iii) a Clinical Dementia Rating (CDR) scale ≤1; (iv) no depression (Montgomery-Asberg Depression Rating Scale (MADRS) score <15); (v) presence of a study partner who is willing to participate as a source of information and has at least weekly contact with the patient. We have chosen not to exclude patients with mild executive dysfunction and social cognition impairment in order to investigate the possible relationship of these clinical features with the underlying aetiology.

Thirty healthy elderly controls (age = 68.8 ± 5.6, MMSE = 29.2 ± 0.9) were recruited according to the following criteria: (i) Mini-Mental State Examination (MMSE) score ≥ 27/30; (ii) normal neuropsychological assessment; (iii) CDR = 0; (iv) no memory complaint; and (v) negative PiB-PET imaging.

Subjects were not included in the study if they had: (i) sleep apnea; (ii) extrapyramidal signs or neurological history suggestive of Parkinson’s disease with dementia, progressive supranuclear palsy, corticobasal degeneration or dementia with Lewy bodies; (iii) systemic illnesses that could interfere with cognitive functioning; (iv) structural abnormality on MRI (intra-parenchymal tumour or large cortical infarct).

### Study design

At baseline, all participants underwent the same procedures including a complete clinical and neuropsychological assessment, 3 T brain MRI, [^11^C]-PiB and [^18^F]-Flortaucipir PET imaging. For controls, [^18^F]-Flortaucipir PET was performed in 13/30 subjects. Participants were then followed up annually with repeated standardized clinical and neuropsychological assessments for 2 years. Thirty-two patients and 30 controls were clinically followed up at one year, and 28 patients and 28 controls at 2 years. At the last visit, subjects were asked to undergo a second 3 T MRI (performed in 27/36 patients and 28/30 controls) and tau PET imaging (performed in 20/36 patients, *n* = 6 refused, *n* = 2 were lost to follow-up; *n* = 8 were outside the regulatory limits of the study). Blood samples were drawn to determine *APOE* and *c9orf72* genotypes as well as plasma progranulin levels in patients.

### Measures

#### Clinical, functional, and cognitive assessment

All subjects underwent a complete clinical and neuropsychological examination that included the Mini-Mental State Examination, the Clinical Dementia Rating scale, tests for assessing verbal (FCSRT) and visual (recall of the Rey complex figure) episodic memory, executive functions (digit spans forward and backward, Trail making test, letter fluency, Similarities subtest of the WAIS III), social cognition (facial emotion recognition and faux-pas subtests of the Mini-SEA) [27], language (word naming task, category fluency), gestural praxis and visuo-spatial abilities (copy of the Rey complex figure). We defined a verbal episodic memory score as the sum of the scores obtained for the free and cued recalls of the FCSRT. Behavioural changes in daily living were assessed by the response provided by an informant to the revised version of the Cambridge Behavioural Inventory [28].

#### Magnetic resonance imaging

All subjects underwent magnetic resonance imaging performed at the Centre de Neuro-Imagerie de Recherche (CENIR, ICM, Paris) using a 3 T whole-body PRISMA 64-channel system (for all patients and 14 controls) or TRIO 32 channels with a 12-channel head coil for signal reception (Siemens) (for the remaining 16 controls). A longitudinal MRI after two years was performed for 27 patients and 28 controls. The MRI examination included a three-dimensional (3D) T1-weighted volumetric magnetization-prepared rapid gradient echo (MP-RAGE) sequence (repetition time/echo time/flip angle: 2300 ms/3.43 ms/9°, inversion time = 900 ms, voxel size: 1 × 1 × 1 mm^3^). Before pooling our control subjects, we verified that the mean cortical thicknesses were not different between both subgroups of controls according to the scan. We used both VOI and vertex-wise complementary methods.

In a hypothesis-driven VOI analysis, we studied gray matter volumes in the following specific regions of interest: (1) the hippocampi and entorhinal cortices, whose atrophy is associated with amnestic syndromes (2) the amygdala, which are affected in non-AD pathologies, especially in LATE [13], (3) the insula, which has also been associated with non-AD progressive amnesia [29]. Volumetric segmentation of the hippocampi, amygdala, entorhinal cortices and insula was automatically performed on the 3D T1-weighted MP-RAGE scans using FreeSurfer 6.0.0 longitudinal processing stream (http://surfer.nmr.mgh.harvard.edu/) [30]. The volume measures were normalized to the individual intracranial volume (IV).

Mean cortical thickness indices for 68 VOIs were also obtained. We visually inspected the FreeSurfer parcellation results to identify global segmentation abnormalities and performed manual edits of the brainmask (pial surface errors) or of the white matter volume (segmentation errors) when necessary.

We also studied cortical thickness without any pre-specified VOI by using FreeSurfer’s built-in statistical tools for vertexwise generalized linear models on the individual surfaces previously resampled into the common anatomic space and smoothed using a Gaussian smoothing kernel of 8 mm. The group comparison (controlled for age) consisted of the following contrasts: controls>AD patients; controls>SNAP patients; AD patients>SNAP patients; SNAP patients> AD patients. Longitudinal data were analysed with a two-stage model by first computing the symmetrized percentage change of cortical thickness and then applying the contrasts mentioned above.

Cluster-wise corrections for multiple comparisons were performed using the permutation-based approach implemented in Freesurfer (number of permutations: 5000, cluster forming threshold: *p* < 0.001). The cluster-wise level of statistical significance was set at *p* < 0.05.

#### [^11^C]-PiB and [^18^F]-Flortaucipir PET imaging procedure

*Data acquisition:* All subjects underwent [^11^C]-PiB PET. All patients (*n* = 36) and 13 controls also underwent [^18^F]-Flortaucipir PET. Twenty patients underwent a second tau PET exam after 2 years. MRI and PET scans were performed within 4 months of each other. All PET examinations were performed at Service Hospitalier Frédéric Joliot (Orsay, CEA) on a High-Resolution Research Tomograph (HRRT; CTI/Siemens Molecular Imaging). PET acquisitions were performed at least 40 to 60 min after injection of 332 ± 60.8 MBq of [^11^C]-PiB, and 80–100 min after injection of 376.2 ± 20.6 MBq of [^18^F]-Flortaucipir.

All post-processing image corrections (attenuation, normalization, random and scatter coincidences) were incorporated in an iterative ordinary Poisson ordered-subset expectation maximization (OP-OSEM) algorithm. Partial volume effect (PVE) was corrected by directly modelling the detector spatial resolution properties (i.e. Point Spread Function modelling) in the image reconstruction algorithm [31, 32], allowing improved spatial resolution and thus reduced PVE without applying a standard partial volume correction technique. Dynamic list mode acquisitions were binned into successive 5-min time frames.

*Volume of interest analysis:* Parametric images were created using BrainVisa software (http://brainvisa.info) on averaged images over 40–60 min after injection of [^11^C]-PiB and over 80–100 min after injection of [^18^F]-Flortaucipir. Standard Uptake Value ratio (SUVr) parametric images were obtained by dividing each voxel by the corresponding value found in the cerebellar gray matter eroded (4 mm) in order to avoid including the superior part of the cerebellar vermis, which is a site of Flortaucipir off-target binding, and to avoid PVE from the CSF or occipital cortex. This structure is used as a reference region, as it has been found to be spared from amyloid plaque and tau accumulation in AD [20, 33, 34] as well as of tau lesions in non-AD tauopathies [35, 36] until the very late stages of disease.

An automated segmentation of the gray matter was performed on the 3D T1 MR images of each subject using SPM8 software (Institute of Neurology, London, UK; http://www.fil.ion.ucl.ac.uk/spm/). Automatic segmentation defined volumes of interest (VOI), which were warped in the T1 space of each subject and intersected with the T1 MRI gray matter mask to perform a pseudo atrophy correction. These VOIs on individual MRI scans were then applied on PET space in each subject after coregistration using a standard mutual information algorithm. The VOIs were segmented using the Automated Anatomic Labeling (AAL) Atlas for cortical structures, FreeSurfer (v 6.0.0.) for the entorhinal cortices, and VolBrain (https://volbrai.upv.es) [37] for the amygdala. The VOIs defined separately for the left and right hemispheres were pooled into greater anatomical volumes of interest.

We considered the following regions for tau PET imaging in order to both possibly detect tau deposition in circumscribed limbic regions and recapitulate key features of Braak progression [21]: entorhinal cortices (stage I/II), amygdala, parahippocampal and fusiform gyri (stage III), inferior and middle temporal gyri, and posterior cingulate (stage IV), superior temporal gyri, frontal, parietal and occipital cortices (stage V), and precentral gyri (stage VI).

### Classification of the subjects according to molecular PET imaging

To define the positivity of amyloid PET imaging, we calculated a [^11^C]-PiB Global Cortical Index (GCI), representing the subject’s mean SUVr of the neocortical regions [33, 38]. The cut-off value of PiB GCI positivity was set as 1.45 [38].

To assess Tau PET binding, we calculated the SUVr in all VOIs (expressed as the mean of the left and right sides) and considered for each region the values above the mean of those obtained in our control group + 1.96 SD as significantly increased (95% confidence interval).

In accordance with the recent biological definition of AD [11], the AD molecular signature was defined as the combination of: (i) positive amyloid PET, and (ii) increased tau PET in the entorhinal cortices and at least one of the following regions: amygdala, parahippocampal gyri, fusiform gyri. The patients who did not fulfil these criteria were considered as having a non-AD pathology (SNAP).

### Statistical analysis

The data were analysed using R version 3.6.1 (R Core Team, 2017). Differences between groups at baseline were assessed using analysis of covariance (ANCOVA), with age and education (for neuropsychological variables) and age and sex (for MRI variables) as covariates, after verifying that the residuals were normally distributed. Longitudinal clinical, MRI and tau PET data were analysed using mixed-effect models accounting for the repeated nature of the measures and for missing data. Subjects were included as a random intercept in the model, and age and education (for clinical data) or age and sex (for MRI data) were included as covariates.

Post-hoc pairwise *t*-tests, adjusted with a Bonferroni correction, were performed to test differences in baseline and longitudinal clinical and imaging parameters between groups. The level of statistical significance was set at *p* < 0.05.

## Results

### Baseline classification and clinical-neuropsychological features of the sample

When combining amyloid and tau PET status as defined above, the 36 patients were divided into 2 subgroups: AD patients (*n* = 21) and SNAP patients (*n* = 15). The SNAP patients were significantly older than the AD patients and controls.

Significant differences were found between each patient subgroup and controls for the MMSE, episodic memory scores, category fluency, Trail Making Test and Faux pas test. There were no statistically significant differences between SNAP and AD patients’ subgroups (Table 1). The individual analysis of the data did not show a clear qualitative difference between SNAP and AD patients, apart from a slight tendency for more abnormal category fluency scores in AD patients (6/21 vs 3/15), and more abnormal facial emotion recognition scores in SNAP patients (6/15 vs 3/21) (Supplementary Table 1).

**Table 1:** Main demographic, clinical, imaging, and biological data at baseline for the patient and control groups (mean (SD)).

				**Whole ASHT group*n*** = 36	**SNAP*n*** = 15	**AD*n*** = 21	**Controls*n*** = 30
Demographic data		Age (years)		72.9 (6.8)	77.5* (5.5)	69.5 (5.6)	68.8 (5.6)
		Sex (F/M)		16/20	6/9	10/11	20/10
		Education (years)		14.3 (4.2)	13.6 (4.7)	14.7 (3.9)	12.7 (4.1)
		Disease duration (years)		4.8 (3.4)	4.2 (2.7)	5 .2 (3.8)	–
Functional status		CDR	0	0	0	0	30
			0.5	26	9	17	0
			1	10	6	4	0
Neuropsychological assessment							
Global cognitive efficiency		MMSE		24.5 (2.8)	24.1 (2.6)	24.8 (2.9)	29.2 (0.9)*
Short term/working memory		Digit spans		9.7 (1.8)	9.9 (2.2)	9.5 (1.6)	10 (1.3)
Episodic memory	verbal	FCSRT (Free + total recall)		33.3 (17)	36 (16.2)	31.4 (17.7)	80.2 (4.5)*
		Free recall (/48)		9.1 (6.3)	9 (5.6)	9.2 (6.8)	32.7 (4)*
		Total recall (/48)		24.2 (11.4)	27 (11.2)	22.1 (11.4)	47.4 (0.9)*
		Sensitivity index (%)		41.1 (21.7)	48.4 (22.2)	35.9 (20.2)	96.7 (5.1)*
	visual	Rey memory (/36)		7.25 (5.1)	7.3 (5.4)	7.2 (5.1)	18.7 (5.5)*
Instrumental functions		Naming (/80)		77.7 (2.8)	78.5 (1.6)	77.1 (3.4)#	79.8 (0.5)
		Rey copy (/36)		34.4 (2.2)	34.7 (2.6)	34.1 (1.9)	35 (1.7)
		Praxis (/72)		69.1 (2.9)	69.6 (1.8)	68.7 (3.4)	71 (1.3)
Executive functions		TMT A (seconds)		53.7 (16.6)	54.5 (17.1)	53.1 (16.6)	39.8 (14.1)
		TMT B-A (seconds)		104.9 (63.3)	106.5 (60.6)	103.7 (66.6)	37.9 (23.6)*
		Letter Fluency (2 min)		18.2 (5.8)	16.1 (5.1)#	19.7 (5.9)	24.6 (7.1)
		Category Fluency (2 min)		20.4 (6.4)	20.9 (7.8)	20 (5.4)	35.5 (9.6)*
		Similarities (WAIS)		19.6 (4.4)	20.7 (3.9)	18.9 (4.7)^#^	22.3 (3.4)
Social cognition		Emotion recognition (/35)		28.5 (3.4)	27.8 (3.3)	29 (3.5)	29.6 (2.3)
		Negative emotions (fear, sadness, anger, disgust) (/20)		14.8 (2.8)	14.3 (2.9)	15.1 (2.7)	15.6 (2)
		Faux pas test (/40)		30.4 (5.2)	30.9 (4.7)	30 (5.7)	35.1 (2.9)*
Behavioral changes		CBI-R (/180)		38 (19.4)	42.1 (19.9)	35.4 (19.1)	–
Genetic status		*ApoE* genotype (n with at least one E4 allele)		18	2	16	2
		Progranulin plasma level (μg/l)		108.5 (26)	114.6 (20.2)	104.1 (29.1)	127.3 (25.5)
		*c9orf72* mutation		None	None	None	–
Molecular PET imaging		PiB PET GCI		2.28 (0.9)	1.35 (0.2)	2.93 (0.6)*	1.25 (0.1)
		n with GCI > 1.45		24	3	21	0
		Tau PET GCI		1.54 (0.65)	1.15 (0.1)	1.81 (0.7)	1.23 (0.11)
MRI		Fazekas score (0/1/2/3)		20/12/3/1	7/5/2/1	13/7/1/0	20/8/2/0
		Left HV		1.78 (0.3)	1.64 (0.27)	1.88 (0.29)	2.48 (0.2)*
		Right HV		1.85 (0.34)	1.68 (0.3)	1.97 (0.32)	2.51 (0.23)*
		Left EC		0.92 (0.32)	0.82 (0.26)	0.99 (0.34)	1.36 (0.23)*
		Right EC		0.93 (0.27)	0.91 (0.3)	0.94 (0.26)#	1.25 (0.23)
		Left Amygdala		0.66 (0.14)	0.64 (0.15)	0.67 (0.13)	0.93 (0.13)*
		Right Amygdala		0.8 (0.16)	0.79 (0.18)	0.82 (0.14)	1.13 (0.15)*
		Left Insula		3.88 (0.42)	3.76 (0.45)	3.97 (0.38)	4.4 (0.47)*
		Right Insula		3.84 (0.5)	3.74 (0.64)	3.92 (0.36)	4.3 (0.46)*

*ASHT* amnestic syndrome of the hippocampal type, *CDR* Clinical Dementia Rating scale, *MMSE* Mini-mental state examination, *FCSRT* Free and Cued Selective Reminding Test, *TMT* Trail Making Test, *WAIS* Wechsler Adult Intelligence Scale, *CBI-R* revised version of the Cambridge Behavioural Inventory, *ApoE* Apolipoprotein E, *PiB-GCI* Pittsburgh compound B global cortical index, *HV* hippocampal volume, *EC* entorhinal cortex. All volumes are normalized to the intracranial volume.

*p < 0.05 vs the other groups ^#^*p* < 0.05 vs controls.

The number of subjects with at least one ε4 allele of the *ApoE* gene was higher in the AD subgroup (Table 1).

### Baseline imaging features of the sample

**Fig. 1:**
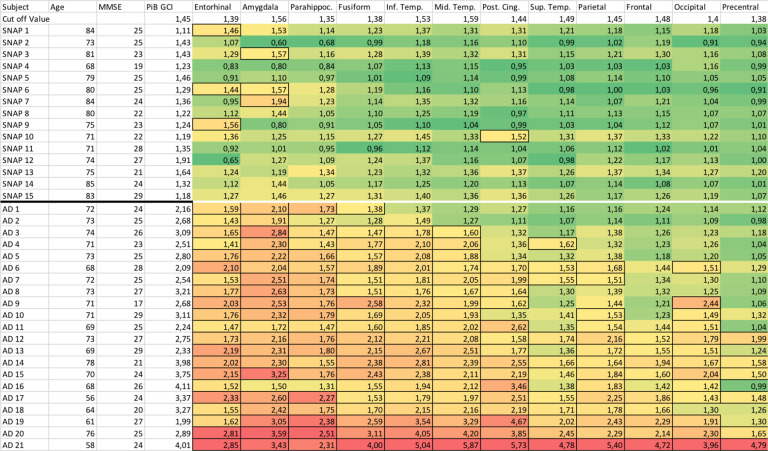
Individual tau PET imaging SUVr (mean of the left and right sides) for each SNAP and AD patient in each VOI. Green boxes correspond to the lowest values and red boxes to the highest values. The boxes are framed when the SUVr is above the cut-off value defined from our control group. MMSE, Mini-mental State Examination; GCI, global cortical index; Parahippoc, parahippocampal gyri; Inf. Temp., Inferior temporal gyri; Mid.Temp., Middle temporal gyri; Post. Cing., posterior cingulate; Sup. Temp., Superior temporal gyri.

Individual PiB GCI and tau PET SUVr in all VOIs are detailed in Fig. 1.

**Fig. 2:**
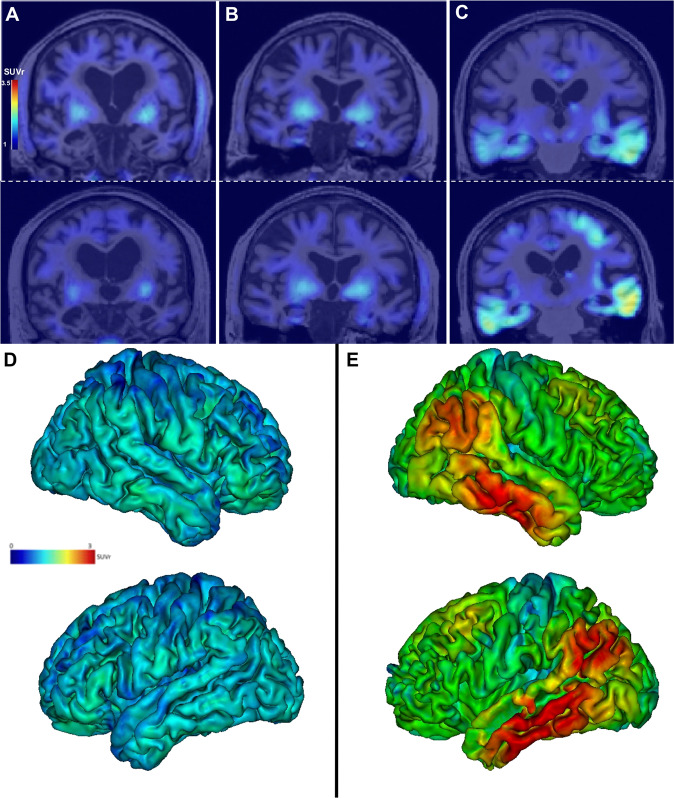
Examples of tau PET images. Individual tau PET SUVr images (coronal sections) in patient SNAP 5 **A**, in patient SNAP 6, who has increased tau radiopharmaceutical uptake in the entorhinal cortices and amygdala with negative amyloid PET (**B**), and in patient AD 9 (**C**) at baseline (top row) and after 2 years (bottom row). Note the off-target binding of the tracer in the basal ganglia in all patients. Projection of the mean cortical [^18^F]-Flortaucipir SUVr on the 3D MRI MNI template in the SNAP group **D** and the AD group **E** (mean images were obtained after normalisation of individual images to the MNI template). Lateral views of the right and left hemispheres are shown.

In AD patients, in addition to the positivity of PiB-PET, tau positivity extended beyond the medial temporal regions. In the SNAP group, PiB-PET was negative in 12/15 patients. The 3 SNAP patients with positive amyloid PET had negative tau PET, including no significant uptake in the entorhinal cortices, amygdala, parahippocampal or fusiform gyri. We found positive tau tracer binding restricted to the entorhinal cortices and/or amygdala in 5 (amyloid negative) SNAP patients (Figs. 1 and 2).

SNAP and AD groups had significantly lower hippocampal, entorhinal, amygdala, and insula volumes than the controls. There was no statistically significant difference between SNAP and AD subgroups (Table 1).

**Fig. 3:**
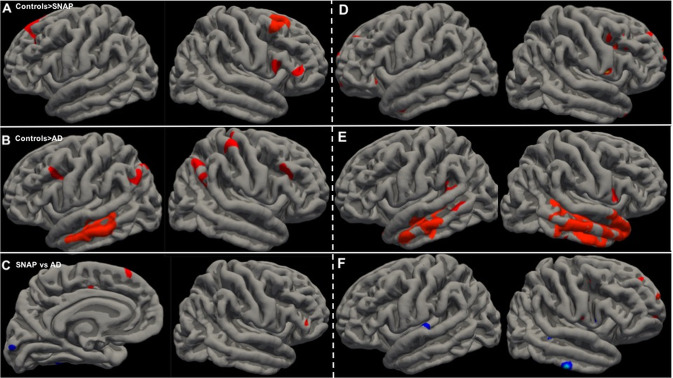
Vertexwise comparisons of cortical thickness between groups. Vertexwise comparison of baseline cortical thickness controlled for age showed lower thickness in: **A** SNAP patients compared with controls (clusterwise correction for multiple comparisons, *p* < 0.05); **B** AD patients compared with controls (clusterwise correction for multiple comparisons, *p* < 0.05); and **C** SNAP patients (in red) and AD patients (in blue) when compared to each other (medial view of the left hemisphere and lateral view of the right hemisphere, *p* < 0.001, uncorrected). Vertexwise comparison of the symmetrized percent change of cortical thickness controlled for age after 2 years showed a higher decrease in: **D** SNAP patients vs controls (*p* < 0.001, uncorrected); **E** AD patients vs controls (clusterwise correction for multiple comparisons, *p* < 0.05); and **F** SNAP patients (in red) and AD patients (in blue) when compared to each other (clusterwise correction for multiple comparisons, *p* < 0.05 in the left hemisphere, and *p* < 0.001, uncorrected in the right hemisphere).

The vertexwise comparison of cortical thickness between patients and controls showed a significant decrease in the frontal lobes (superior and middle frontal gyri) in SNAP patients and in the temporo-parietal and frontal lobes in AD patients. The direct comparison between SNAP and AD patients showed lower (uncorrected) baseline cortical thickness in the frontal cortex in SNAP patients and in the posterior (temporal and occipital) cortex in AD patients (Fig. 3).

### Longitudinal clinical-neuropsychological analysis

**Fig. 4:**
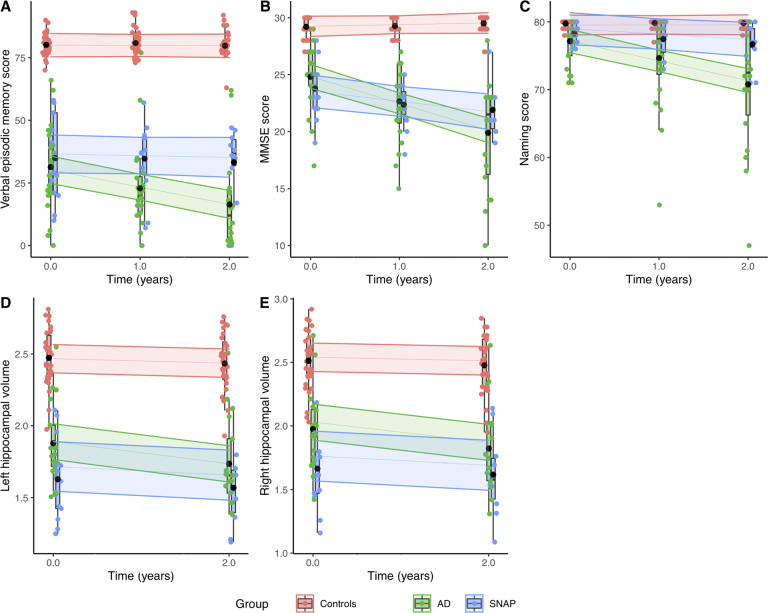
Evolution of the cognitive scores and hippocampal atrophy. Temporal diagrams representing the evolution of the verbal episodic memory score (sum of the free and cued recalls of the FCSRT) (**A**), MMSE score (**B**), and naming score (**C**) at baseline and after 1 and 2 years of follow-up and the evolution of the left (**D**) and right (**E**) hippocampal volumes at baseline and after 2 years of follow-up in each patients’ group and controls. The ribbons represent the 95% confidence interval. Boxplots with individual points for each group are shown. The black point represents the mean value for each group.

The decrease of the MMSE, verbal episodic memory, and naming scores was significantly higher in AD (*n* = 20 at one year and 18 at 2 years) than in SNAP (*n* = 12 at one year and 10 at 2 years) patients (*p* = 0.04, 0.01, and 0.03, respectively) (Fig. 4). There were no other significant differences. The individual analysis of the data also showed a higher number of patients whose naming score became abnormal during follow-up in the AD group (9/20 vs 2/12). We also found the same trend for letter fluency (6/20 vs 2/12) and the similarities subtest of the WAIS (5/20 vs 1/12). There was no difference regarding social cognition scores (Supplementary Table 1).

### Longitudinal MRI analysis

The decrease of hippocampal volumes was higher in AD (*n* = 17) than in SNAP (*n* = 10) patients (*p* = 0.007 for the right hippocampus and *p* = 0.025 on the left side) (Fig. 4). There was no significant difference regarding the progression of entorhinal, amygdala or insula atrophy.

The vertexwise comparison of the symmetrized percent change of cortical thickness after 2 years showed a significant decrease in the temporal lobes for AD patients compared with controls (*n* = 28). The (uncorrected) comparison between SNAP patients and controls showed spots of decreased cortical thickness in the frontal lobes. The comparison between AD and SNAP patients showed a cluster of decreased cortical thickness in the left superior temporal gyrus in AD patients and (uncorrected) spots in the frontal lobes in SNAP patients (Fig. 3).

### Longitudinal tau PET imaging

**Fig. 5:**
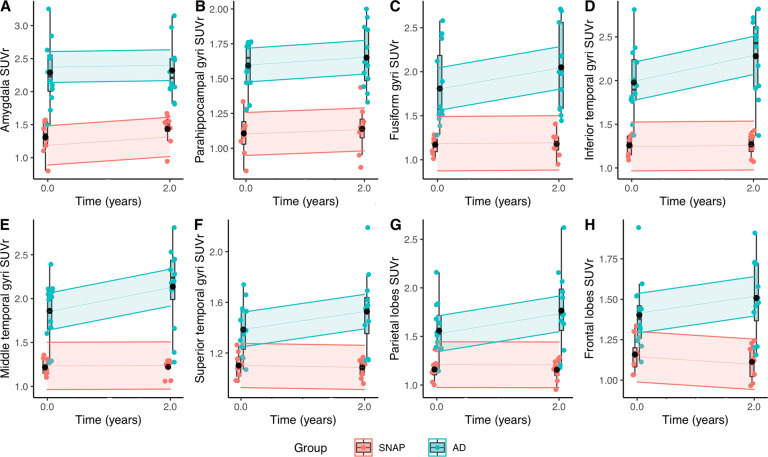
Evolution of the tau tracer binding. Temporal diagrams representing the evolution of the tau PET SUVr in the amygdala (**A**), parahippocampal gyri (**B**), fusiform gyri (**C**), inferior temporal gyri (**D**), middle temporal gyri (**E**), superior temporal gyri (**F**), parietal lobes (**G**) and frontal lobes (**H**) at baseline and after 2 years of follow-up in the SNAP and AD groups. The ribbons represent the 95% confidence interval. Boxplots with individual points for each group are shown. The black point represents the mean value for each group.

Eight SNAP patients and 12 AD patients underwent a second tau PET exam after 2 years. Tau tracer binding remained stable in all VOIs in SNAP patients, even in those with initially elevated binding in the entorhinal cortices and amygdala, while it globally increased in AD patients. We found a significantly higher increase of tracer binding in AD patients than in SNAP patients in the middle temporal gyri (*p* = 0.04) and in the parietal lobes (*p* = 0.025). We also found differences in the fusiform (*p* = 0.026), as well as inferior (*p* = 0.008), and superior (*p* = 0.01) temporal gyri and in the frontal lobes (*p* = 0.03), which did not persist after correction for multiple comparisons (Fig. 5, Supplementary Table 2).

## Discussion

By coupling amyloid and tau PET imaging in a clinically homogeneous cohort of 36 patients with ASHT, we aimed to identify AD and SNAP patients according to their molecular signatures and investigate their clinical and imaging trajectories after 2 years of follow-up.

We found that: (i) an important proportion of patients did not meet the molecular definition of AD and were thus classified as SNAP, with some of them having tau tracer binding limited to the entorhinal cortices and/or amygdala without amyloid deposition; (ii) SNAP patients were older and showed a lower *ApoE* ε4 allele frequency than AD patients but both groups had comparable isolated ASHT and a similar degree of medial temporal atrophy at baseline; (iii) disease progression differed in SNAP and AD patients regarding cognitive profile, brain atrophy and tau deposition patterns.

Only two studies with small sample sizes have so far explored the in vivo molecular signature of amnestic patients using both amyloid and tau PET imaging [24, 25]. Contrary to our work, the authors studied tau binding in a unique temporal meta-VOI, which includes entorhinal, parahippocampal, fusiform cortices and inferior and middle temporal gyri (defined by Jack et al. [39]). Using both amyloid (global cortical index) and tau positivity in the temporal meta-VOI as a molecular definition of AD, 62% (15/24) of patients met the criteria for AD. As they were tau negative in the temporal meta-VOI, the other patients were considered as SNAP by the authors despite positive amyloid PET in some of them. Nevertheless, we cannot rule out a circumscribed tauopathy in the limbic regions suggestive of AD in these amyloid-positive amnestic patients. In addition, no longitudinal data were available.

In order to avoid any misinterpretation in the assessment of tau tracer uptake, we preferred to measure tau binding in selective regions corresponding to the earliest Braak stages within the medial temporal lobes rather than considering only one larger temporal meta-VOI. By doing this, we aim at identifying circumscribed tau deposits in areas involved in episodic memory both in amyloid-positive (early AD) and amyloid-negative (SNAP) patients.

In addition, we used strict inclusion criteria, i.e., ASHT without any other neurological or psychiatric signs, to avoid bias in the interpretation of the results. By investigating patients without an AD signature, we aimed to explore limbic predominant non-Alzheimer’s pathology, which is one major source of misdiagnosis in ASHT [9, 24].

We found that 42% of our patients with ASHT did not have an AD molecular signature and were therefore classified as SNAP. This result is consistent with neuropathological and previous imaging studies showing that an ASHT mimicking AD can be found without any amyloid or tau lesions [23, 24, 40]. In these conditions, the most frequent underlying pathologies are hippocampal sclerosis of ageing (most often associated with TDP-43 lesions), LATE neuropathological change (LATE-NC), and PART. The other possible aetiologies, such as Lewy body dementia, are less likely due to our strict clinical inclusion criteria.

LATE-NC is defined by a stereotypical TDP-43 pathology with or without hippocampal sclerosis [12, 13, 25]. The estimated prevalence reaches 30% among subjects autopsied past 80 years of age and increases with advanced old age [12, 13]. Interestingly, in our cohort, SNAP patients were indeed older than AD patients. Many subjects with LATE-NC have comorbid brain pathologies, often including amyloid plaques, which could be the case in our 3 amyloid-positive SNAP patients [13]. In general, the co-occurrence of different brain lesions, whether of the AD type, other proteinopathies (particularly TDP-43 or alpha-synuclein, which are often associated with AD), or vascular lesions, is a frequent finding in aged samples [41] and further complicates the in vivo characterization of the aetiology. In addition, the diagnosis of LATE is difficult to confirm in the absence of TDP-43-specific biomarkers [13].

In a subset of SNAP patients (*n* = 5/15), we observed a circumscribed tau tracer retention in the entorhinal cortices and amygdala, clearly distinct from the more extensive pattern of tau binding observed in AD. Even if the sensitivity of Flortaucipir for non-AD tauopathies has been questioned, elevated tracer retention compared to controls has been found in areas with non-Alzheimer tau pathology at autopsy [42]. This pattern of restricted tau tracer binding in the medial temporal cortex in the absence of amyloid pathology could therefore be suggestive of PART [43] a recently defined neuropathological category characterized by tau aggregates and neuronal loss in the medial temporal lobes without amyloid plaques [14]. In autopsy studies, PART occurs in 20–25% of individuals older than 90 years [16]. The cognitive presentation seems variable and remains under discussion [16]. Our results suggest that PART could be more frequent than previously thought before the age of 90 and could lead to ASHT.

The specificity of the tau PET radiopharmaceuticals has been discussed. Some Flortaucipir binding to the TDP-43 protein underlying FTD has been described in semantic dementia [44]. However, our amyloid-negative patients with increased tau binding had no semantic deficit, and binding to the TDP-43 protein underlying FTD would extend beyond the medial temporal lobes, which was not the case. Off-target binding, as observed in the basal ganglia or the hippocampi due to their close vicinity to the choroid plexus, is unlikely in the regions we have considered.

At baseline, SNAP and AD patients had identical cognitive profiles characterized by an isolated ASHT and a similar degree of medial temporal lobe atrophy, as previously described in HS and LATE with autopsy confirmation [12, 13]. We did not find any neuropsychological difference between both subgroups, apart from a qualitative trend towards more abnormal facial emotion recognition scores in the SNAP group, which was not confirmed for the faux-pas test and therefore does not clearly point to a characteristic social cognition deficit in these patients, as has been described recently [45]. Medial temporal lobe atrophy was slightly more pronounced in SNAP patients, in accordance with previous publications, perhaps due to their older age [5, 13]. This was not the case only in the hippocampi but also in the entorhinal cortices and amygdala, which has been thought to represent an “incubator” for misfolded proteins [46]. Nevertheless, we found differences in the cortical atrophy patterns between SNAP and AD, with predominant frontal involvement in SNAP, as has been reported in hippocampal sclerosis and TDP-43 proteinopathies [13, 47–50], and predominant temporo-parietal involvement in AD, as expected.

After 2 years of follow-up, we observed distinct cognitive trajectories, with greater episodic memory and language decline in AD than in SNAP. Even if the cognitive trajectories in our SNAP patients were less homogeneous than in the AD group, probably due to heterogeneous underlying mechanisms, our results are in accordance with previous work on PART and HS [15–17, 49, 51]. We did not find a more pronounced decline in executive functions or social cognition in SNAP patients, contrary to what might have been expected [45].

The longitudinal MR imaging analysis showed a greater progression of hippocampal atrophy in AD than in SNAP, which was not reported in a previous work that found a similar atrophy progression [15]. However, in the latter study, the patient classification was performed without tau-PET imaging. In our cohort, the decline of cortical thickness also differed between AD and SNAP patients, with pronounced changes in the temporal lobes in AD patients and a more subtle decrease predominating in the frontal cortex in SNAP patients.

The longitudinal tau PET imaging after 2 years reinforces the distinction between the AD and SNAP groups by showing an increase of tau binding in AD patients, especially in the parietal and temporal cortices, which contrasts with the stability of tau binding in SNAP patients, including those with positive PiB-PET. Circumscribed medial temporal tau tracer binding was confirmed in the patients with suspected PART who underwent longitudinal tau-PET, without any cortical extension. This is in agreement with a non-AD diagnosis and with the slower disease progression observed [51].

Our study has several limitations, especially the relatively small sample size with missing data in the longitudinal analysis, and the lack of neuropathological confirmation. However, the longitudinal clinical and imaging data strengthen the distinction between AD and non-AD pathological processes. The small numbers of subjects in the different supposed SNAP subgroups make it difficult to account for subtle differences related to the clinical heterogeneity of this entity, which could help to tease apart the different aetiologies of SNAP. The presence and potential role of copathologies in AD patients is also difficult to determine in the present cohort, although this probably has important consequences on the clinical presentation and cognitive progression.

Following the description of HS of ageing, LATE-NC and PART, it appears necessary to increase our understanding of the pathophysiological processes leading to ASHT in order to better target new disease modifying therapies. By coupling amyloid and tau-PET with longitudinal follow-up, we found distinct in vivo molecular signatures, which have consequences on clinical and atrophy progression. Longitudinal clinical and PET imaging data in larger cohorts will be important to improve our ability to determine the in vivo molecular diagnosis of this misleading cognitive profile, which is the only way to develop curative treatments.

## Supplementary information

Supplementary table 1

Supplementary table 2
