# Supplementary material for: Distinct amyloid and tau PET signatures are associated with diverging clinical and imaging trajectories in patients with amnestic syndrome of the hippocampal type
Source: Transl Psychiatry. 2021 Sep 29;11:498. doi: 10.1038/s41398-021-01628-9 (PMC8481505; doi:10.1038/s41398-021-01628-9)
Supplement: Supplementary file 1 — Supplementary table 1 [file 41398_2021_1628_MOESM1_ESM.docx]

| Subject | AT | Age | Sex | Educ (y) | Dis. Dur. (y) | APOE | MMSE | | | CF | | | LF | | | Simil. | | | FER | | | Faux Pas | | | Naming | | | Rey copy | | | Praxis | | |
| --- | --- | --- | --- | --- | --- | --- | --- | --- | --- | --- | --- | --- | --- | --- | --- | --- | --- | --- | --- | --- | --- | --- | --- | --- | --- | --- | --- | --- | --- | --- | --- | --- | --- |
|  |  |  |  |  |  |  | B | 1 | 2 | B | 1 | 2 | B | 1 | 2 | B | 1 | 2 | B | 1 | 2 | B | 1 | 2 | B | 1 | 2 | B | 1 | 2 | B | 1 | 2 |
| SNAP 1 | A-T+ | 84 | M | 17 | 2 | 3/4 | 25 | 23 | 24 | U | I | I | U | U | I | U | U | U | U | U | I | U | U | U | U | U | U | U | U | U | U | U | U |
| SNAP 2 | A-T- | 73 | M | 18 | 4 | 3/3 | 25 | 22 | 20 | I | I | I | I | I | I | U | U | I | I | I |  | U | I |  | U | U | I | U | U | U | U | U | U |
| SNAP 3 | A-T+ | 81 | F | 15 | 2 | 4/4 | 23 | 20 | 21 | I | I | I | U | U | U | U | U | U | U | U | U | I | U | U | U | U | U | U | U | U | U | U | U |
| SNAP 4 | A-T- | 68 | F | 11 | 4 | 3/3 | 19 | 22 | 23 | U | U | U | U | U | U | U | U | U | U | U | U | I | U | U | U | U | U | U | U | U | U | U | U |
| SNAP 5 | A+T- | 79 | M | 12 | 3 | 2/3 | 25 | 18 | 19 | U | I | I | I | I | I | U | U | U | U | I | I | I | I | I | U | U | U | U | U | U | U | I | I |
| SNAP 6 | A-T+ | 80 | M | 16 | 4 | 3/3 | 25 | 25 | 23 | U | U | U | U | U | U | U | U | U | I | U | U | I | U | I | U | U | U | U | U | U | U | U | U |
| SNAP 7 | A-T+ | 84 | M | 15 | 7 | 3/3 | 24 | 25 | 21 | U | I | I | U | U | U | U | U | U | U | U | U | U | U | U | U | U | I | U | U | U | U | U | U |
| SNAP 8 | A-T- | 80 | F | 18 | 2 | 3/3 | 22 | 23 | 21 | I | U | I | I | U | U | U | U | U | I | U | U | U | U |  | U | U | U | U | U | U | U | U | U |
| SNAP 9 | A-T+ | 75 | F | 12 | 1 | 3/3 | 23 | 23 |  | U | I |  | I | I |  | U | U |  | I | U |  | U | I |  | U | U |  | U | U |  | U | U |  |
| SNAP 10 | A-T- | 71 | M | 19 | 6 | 3/3 | 22 | 22 |  | U | U |  | U | U |  | U | U |  | U | U |  | I | U |  | U | U |  | U | U |  | U | U |  |
| SNAP 11 | A-T- | 71 | F | 8 | 5 | 3/3 | 28 |  |  | U |  |  | U |  |  | U |  |  | I |  |  | U |  |  | U |  |  | U |  |  | U |  |  |
| SNAP 12 | A+T- | 74 | M | 14 | 12 | 3/3 | 27 | 24 | 27 | U | U | I | U | U | I | U | U | U | U | U | U | I | U | U | U | U | U | U | U | U | U | U | U |
| SNAP 13 | A+T- | 75 | F | 5 | 3 | 3/3 | 21 | 21 | 20 | U | U | U | U | U | U | U | U | U | U | U | I | I | U | U | U | U | U | U | U | U | U | U | U |
| SNAP 14 | A-T- | 85 | M | 5 | 5 | 3/3 | 24 |  |  | U |  |  | U |  |  | U |  |  | I |  |  | U |  |  | U |  |  | U |  |  | U |  |  |
| SNAP 15 | A-T- | 83 | M | 19 | 3 | 3/3 | 29 |  |  | U |  |  | U |  |  | U |  |  | U |  |  | U |  |  | U |  |  | U |  |  | U |  |  |
| AD 1 | A+T+ | 72 | F | 10 | 3 | 3/4 | 24 | 20 | 22 | U | U | I | U | U | U | U | U | I | I | I | I |  | U | I | U | U | I | U | U | U | U | U | U |
| AD 2 | A+T+ | 73 | M | 17 | 5 | 3/4 | 25 | 24 | 23 | I | I | I | U | I | I | U | I | I | U | U | U | I | U |  | U | U | I | U | U | U | U | I | I |
| AD 3 | A+T+ | 74 | M | 20 | 5 | 3/4 | 26 | 26 | 21 | U | I | I | U | U | I | U | U | U | U | U | U | U | U | U | U | U | I | U | U | U | U | U | U |
| AD 4 | A+T+ | 71 | M | 17 | 6 | 4/4 | 23 | 22 | 20 | U | U | I | U | U | I | U | U | U | U | U | I | I | I | I | U | I | I | U | U | U | U | U | U |
| AD 5 | A+T+ | 73 | M | 20 | 7 | 3/4 | 25 | 24 |  | U | U |  | U | U |  | I | U |  | U | U |  | U | U |  | U | U |  | U | U |  | U | U |  |
| AD 6 | A+T+ | 68 | M | 14 | 3 | 3/4 | 28 | 29 | 27 | U | U | U | U | U | U | U | U | U | U | U | U | I | U | U | U | U | U | U | U | U | U | U | U |
| AD 7 | A+T+ | 72 | M | 14 | 7 | 4/4 | 25 | 21 | 22 | I | U | I | I | I | I | I | U | U | U | U | U | U | I | U | U | U | U | U | U | U | U | I | U |
| AD 8 | A+T+ | 73 | F | 12 | 5 | 3/4 | 27 | 26 | 28 | U | U | U | U | U | U | U | U | U | U | U | U | U | U | U | U | U | U | U | U | U | U | U | U |
| AD 9 | A+T+ | 71 | F | 15 | 6 | 3/4 | 17 | 17 | 14 | I | U | I | U | U | U | U | U | U | U | U | U | I | I |  | U | U | I | U | U | U | U | U | I |
| AD 10 | A+T+ | 71 | F | 16 | 1 | 3/4 | 29 | 27 |  | U | U |  | U | U |  | U | U |  | U | U |  | U | U |  | U | U |  | U | U |  | U | U |  |
| AD 11 | A+T+ | 69 | F | 6 | 3 | 3/4 | 25 | 21 | 24 | U | U | U | U | U | U | U | U | U | U | U | U | U | U | U | U | U | U | U | U | U | U | U | U |
| AD 12 | A+T+ | 73 | F | 20 | 4 | 3/4 | 27 | 23 | 23 | U | U | U | U | U | U | U | U | U | U | U | U | U | U | U | U | U | U | U | U | U | U | U | I |
| AD 13 | A+T+ | 69 | M | 17 | 5 | 3/3 | 29 | 26 | 23 | I | I | I | U | U | U | U | U | U | U | U | U | U | I | U | U | I | I | U | U | U | U | U | U |
| AD 14 | A+T+ | 78 | M | 12 | 20 | 3/4 | 21 | 22 | 16 | U | I | I | I | U | I | U | U | U | I | I | I | I | I |  | U | I | I | U | U | U | U | U | U |
| AD 15 | A+T+ | 70 | F | 10 | 2 | 3/4 | 24 | 19 | 23 | U | U | U | U | U | U | U | U | U | U | U | U | U | U | U | U | U | U | U | U | U | U | U | U |
| AD 16 | A+T+ | 68 | F | 14 | 8 | 3/4 | 26 | 26 | 17 | U | I | I | U | I | U | U | U | U | U | U | U | U | U | U | U | U | U | U | U | U | U | U | U |
| AD 17 | A+T+ | 56 | M | 12 | 4 | 3/3 | 24 |  |  | I |  |  | U |  |  | U |  |  | U |  |  | U |  |  | U |  |  | U |  |  | U |  |  |
| AD 18 | A+T+ | 64 | F | 15 | 5 | 3/3 | 20 | 18 | 10 | I | I | I | U | U | I | I | I | I | U | I | U | I |  |  | U | I | I | U | U | U | U | I | U |
| AD 19 | A+T+ | 61 | M | 19 | 3 | 2/3 | 27 | 25 | 13 | U | U | I | I | I | I | U | I | I | U | I | I | I | I |  | U | U | U | U | U | U | U | U | U |
| AD 20 | A+T+ | 76 | F | 10 | 6 | 3/3 | 25 | 15 | 14 | U | I | I | U | U | I | U | U | I | I | U | U | I |  |  | U | I | I | U | U | U | U | I | U |
| AD 21 | A+T+ | 58 | M | 19 | 2 | 3/3 | 24 | 22 | 18 | U | U | I | U | I | I | U | U | I | U | I | I | I | I |  | U | U | U | U | I |  | U | I | I |

Supplementary table 1

Single case table representing individual demographic and baseline/follow-up neuropsychological variables.

Abbrevations: AT, AT classification; A+, amyloid positive (PiB-GCI>1.45); T+, tau positive (increased tau tracer uptake in at least one VOI); Educ, Education; Dis. Dur, disease duration; y, years; APOE, Apolipoprotein E genotype; MMSE, Mini Mental Test Examination; CF, Category fluency; LF, Letter fluency; Simil, Similarities subtest of the WAIS; FER, facial emotion recognition; B, assessment at baseline; 1, assessment at 1 year; 2, assessment at 2 years; U, unimpaired; I, impaired.

The impaired/unimpaired classification was performed based on the normalized scoring used in clinical practice for each test by taking age, sex and education into account.

Impaired performances at baseline are highlighted in blue. Initially normal scores becoming abnormal during follow-up are highlighted in red.
