# Supplementary material for: Distinct amyloid and tau PET signatures are associated with diverging clinical and imaging trajectories in patients with amnestic syndrome of the hippocampal type
Source: Transl Psychiatry. 2021 Sep 29;11:498. doi: 10.1038/s41398-021-01628-9 (PMC8481505; doi:10.1038/s41398-021-01628-9)
Supplement: Supplementary file 2 — Supplementary table 2 [file 41398_2021_1628_MOESM2_ESM.docx]

| Subject | Visit | Amygdala | Parahippoc. | Fusiform | Inf. Temp | Mid. Temp | Sup. Temp | Parietal | Frontal | Occipital |
| --- | --- | --- | --- | --- | --- | --- | --- | --- | --- | --- |
| SNAP 1 | 1 | 1.53 | 1.14 | 1.23 | 1.37 | 1.31 | 1.21 | 1.18 | 1.15 | 1.18 |
|  | 2 | 1.45 | 1.15 | 1.26 | 1.39 | 1.29 | 1.13 | 1.12 | 1.12 | 1.15 |
| SNAP 3 | 1 | 1.57 | 1.16 | 1.28 | 1.39 | 1.32 | 1.15 | 1.21 | 1.30 | 1.16 |
|  | 2 | 1.53 | 1.12 | 1.26 | 1.34 | 1.29 | 1.11 | 1.16 | 1.23 | 1.19 |
| SNAP 4 | 1 | 0.80 | 0.84 | 1.07 | 1.13 | 1.15 | 1.03 | 1.03 | 1.03 | 1.16 |
|  | 2 | 0.95 | 0.95 | 1.14 | 1.22 | 1.25 | 1.14 | 1.20 | 1.04 | 1.18 |
| SNAP 5 | 1 | 1.10 | 0.97 | 1.01 | 1.09 | 1.14 | 1.08 | 1.14 | 1.10 | 1.05 |
|  | 2 | 1.25 | 0.86 | 0.95 | 1.07 | 1.06 | 0.96 | 1.04 | 0.97 | 0.98 |
| SNAP 6 | 1 | 1.57 | 1.28 | 1.19 | 1.16 | 1.10 | 0.98 | 1.00 | 1.03 | 0.96 |
|  | 2 | 1.63 | 1.15 | 1.05 | 1.09 | 1.06 | 1.00 | 0.95 | 0.98 | 0.90 |
| SNAP 8 | 1 | 1.44 | 1.05 | 1.10 | 1.25 | 1.19 | 1.11 | 1.13 | 1.15 | 1.07 |
|  | 2 | 1.55 | 1.19 | 1.13 | 1.25 | 1.27 | 1.14 | 1.24 | 1.13 | 1.17 |
| SNAP 12 | 1 | 1.27 | 1.09 | 1.24 | 1.37 | 1.16 | 0.98 | 1.22 | 1.17 | 1.13 |
|  | 2 | 1.67 | 1.26 | 1.25 | 1.43 | 1.26 | 1.04 | 1.28 | 1.19 | 1.20 |
| SNAP 13 | 1 | 1.19 | 1.34 | 1.23 | 1.32 | 1.36 | 1.26 | 1.37 | 1.34 | 1.27 |
|  | 2 | 1.47 | 1.44 | 1.41 | 1.36 | 1.29 | 1.17 | 1.26 | 1.24 | 1.24 |
| AD 1 | 1 | 2.10 | 1.73 | 1.38 | 1.37 | 1.29 | 1.16 | 1.16 | 1.24 | 1.14 |
|  | 2 | 2.05 | 1.72 | 1.44 | 1.37 | 1.28 | 1.15 | 1.18 | 1.20 | 1.18 |
| AD 2 | 1 | 1.91 | 1.27 | 1.28 | 1.49 | 1.27 | 1.07 | 1.14 | 1.11 | 1.09 |
|  | 2 | 2.50 | 1.44 | 1.57 | 1.78 | 1.39 | 1.14 | 1.21 | 1.16 | 1.18 |
| AD 3 | 1 | 2.84 | 1.47 | 1.47 | 1.78 | 1.60 | 1.17 | 1.38 | 1.26 | 1.23 |
|  | 2 | 2.07 | 1.39 | 1.50 | 1.91 | 1.66 | 1.15 | 1.36 | 1.21 | 1.16 |
| AD 6 | 1 | 2.04 | 1.57 | 1.89 | 2.01 | 1.74 | 1.53 | 1.68 | 1.44 | 1.51 |
|  | 2 | 2.48 | 1.85 | 2.56 | 2.72 | 2.20 | 1.82 | 1.96 | 1.51 | 1.75 |
| AD 7 | 1 | 2.51 | 1.74 | 1.51 | 1.81 | 2.05 | 1.55 | 1.51 | 1.34 | 1.30 |
|  | 2 | 2.3 | 1.54 | 1.59 | 1.94 | 2.18 | 1.57 | 1.63 | 1.42 | 1.31 |
| AD 8 | 1 | 2.63 | 1.73 | 1.51 | 1.76 | 1.67 | 1.30 | 1.39 | 1.32 | 1.25 |
|  | 2 | 3.15 | 1.94 | 1.80 | 2.17 | 2.10 | 1.45 | 1.66 | 1.48 | 1.55 |
| AD 9 | 1 | 2.53 | 1.76 | 2.58 | 2.32 | 1.99 | 1.25 | 1.44 | 1.21 | 2.44 |
|  | 2 | 2.12 | 1.50 | 2.56 | 2.52 | 2.28 | 1.42 | 1.73 | 1.46 | 2.59 |
| AD 11 | 1 | 1.72 | 1.47 | 1.60 | 1.85 | 2.02 | 1.35 | 1.54 | 1.44 | 1.51 |
|  | 2 | 1.83 | 1.62 | 1.98 | 2.39 | 2.45 | 1.55 | 1.90 | 1.72 | 1.87 |
| AD 12 | 1 | 2.16 | 1.76 | 2.12 | 2.21 | 2.08 | 1.74 | 2.16 | 1.52 | 1.79 |
|  | 2 | 2.51 | 2.00 | 2.69 | 2.82 | 2.81 | 2.19 | 2.62 | 1.91 | 2.26 |
| AD 14 | 1 | 2.30 | 1.55 | 2.38 | 2.81 | 2.39 | 1.66 | 1.64 | 1.94 | 1.67 |
|  | 2 | 2.04 | 1.33 | 2.18 | 2.60 | 2.33 | 1.62 | 1.72 | 1.73 | 1.71 |
| AD 15 | 1 | 3.25 | 1.76 | 2.43 | 2.38 | 2.11 | 1.46 | 1.84 | 1.60 | 2.04 |
|  | 2 | 2.97 | 1.87 | 2.71 | 2.67 | 2.43 | 1.69 | 2.07 | 1.58 | 2.43 |
| AD 16 | 1 | 1.50 | 1.31 | 1.55 | 1.94 | 2.12 | 1.38 | 1.83 | 1.42 | 1.42 |
|  | 2 | 1.81 | 1.59 | 2.00 | 2.47 | 2.53 | 1.57 | 2.16 | 1.73 | 1.70 |

Supplementary table 2

SUVr in each VOI at baseline (Visit1) and after 2 years (Visit 2) for the patients who performed a second tau PET imaging.

Abbreviations: Parahippoc., parahippocampal gyri; Inf. Temp, inferior temporal gyri; Mid.Temp, middle temporal gyri; Sup. Temp, superior temporal gyri.
